# Why my disease is important: metrics of disease occurrence used in the introductory sections of papers in three leading general medical journals in 1993 and 2003

**DOI:** 10.1186/1478-7954-9-14

**Published:** 2011-05-23

**Authors:** Hebe N Gouda, John W Powles

**Affiliations:** 1 Institute of Public Health, Forvie Site, University of Cambridge, CB2 0SP, UK

## Abstract

**Background:**

We assessed the metrics used in claims about disease importance made in the introductory sections of scientific papers published in 1993 and 2003. We were interested in the choice of metric in circumstances where establishing the relative social importance of a disease was, presumptively, a primary objective.

**Methods:**

This study consisted of a textual examination of the introductory statements from papers retrieved from MEDLINE. Papers were published in the *New England Journal of Medicine*, *The Lancet*, and the *Journal of the American Medical Association *during the first halves of 1993 and 2003, and were selected on the basis of keywords found in a pilot study to be associated with claims about disease importance.

**Results:**

We found 143 papers in 1993 and 264 papers in 2003 included claims about disease importance in their introductory sections, and characteristics of these claims were abstracted. Of the quotes identified in the papers and articles examined, most used counts, prevalence, or incidence measurements. Some also used risk estimates and economic quantities to convey the importance of the disease. There was no change in the types of metrics used between 1993 and 2003. Very few articles, even in 2003, used metrics that weighted disease onsets by the expected consequent loss of healthy time -- such as years of life lost, quality-adjusted life years, and/or disability-adjusted life years.

**Conclusions:**

Claims about the relative importance of diseases continued to be overwhelmingly expressed in terms of counts (of deaths and disease onsets) and comparisons of counts, rates, and risks. Where the aim is to convey the burden that a given disease imposes on a society, "event-based" metrics might be less fit for the purpose than "time-based" metrics. More attention is needed to how the choice of metric should relate to the purpose at hand.

## Background

Ranking diseases by their social importance is a central analytic task in health policymaking. Of the many ways in which the occurrence of disease may be measured and expressed, some are more suitable for this purpose than others. Although measures based on disease onsets ("event-based measures") may be optimal for scientific purposes, measures of the consequences of disease onsets generally serve better as measures of the burden that the disease imposes on society. Measures of disease burden are therefore typically expressed in life or healthy time lost. An example, using only mortality data, is the metric of "years of life lost" [1,2].

The 1990s saw a new wave of interest in measures designed to capture the social losses consequent to disease onset. In particular, the ambitious Global Burden of Disease (GBD) initiative devised the disability-adjusted life year (DALY), which first came to general notice in the 1993 *World Development Report*. We were interested to know whether the introduction of the DALY and the growing literature on population health metrics had influenced the choice of metrics used to express the relative social importance of diseases.

In the introduction section of research reports, researchers commonly justify their research topic by making claims about the social importance of the disease under consideration. We have therefore searched for these claims of disease importance in a sample of papers drawn from three leading medical journals in two periods spanning the introduction of the DALY. Further analysis was conducted to assess the extent of the usage of the DALY in the scientific literature indexed on MEDLINE over a longer time period.

## Methods

We selected three high-impact general medical journals: the *New England Journal of Medicine*, the *Journal of the American Medical Association*, and *The Lancet*. We conducted a pilot study to identify search terms that would be likely to be included in papers with claims of interest. We chose the study periods Jan. 1, 1993 to June 30, 1993 and Jan. 1, 2003 to June 30, 2003 to straddle the period of the introduction of new examples of time-based metrics - notably the DALY. Our search in PubMed was limited to original contributions, articles, reviews, and editorials dealing with humans, and article type was restricted to clinical trial, editorial, meta-analysis, review, classical article, comparative study, and journal article.

We downloaded all hits to Endnote for further exploration. First, a search for keywords in Endnote was conducted to identify papers likely to include claims of interest. Keywords included epidemiology, statistics and numerical data, morbidity, mortality, risk, prevalence, incidence, trial (type of work), review (type of work), and etiology. Keywords used to filter out papers unlikely to contain claims of interest included: patient satisfaction, conflict of interest, confidentiality, health services, organization and administration, ethical committee, biomedical research, drug industry, legislation and jurisprudence, malpractice, medical error, case report, altruism, empathy, medical records, physician-patient relations, Hippocratic oath, criminal law, abstracting and indexing, wit and humor, attitude to health, biography, historical article, and social control. The titles of the remaining articles were scanned to identify papers that were focused on specific diseases. Claims about the importance of a disease were extracted and analyzed. We distinguished three main groups of metrics:

*1. Objective *(excluding time-based). This included counts, proportions, risks, and rates. Additionally, we included rankings based on these metrics, e.g., "X is the second leading cause of death"; "Anxiety and panic disorders are approximately twice as frequent among women as among men."

*2. Economic*. This included measures when disease occurrence was expressed in terms of cost or utility (derived from the theoretical framework of economics) but was not time-based, e.g., direct treatment costs.

*3. Time-based*. This class of measures was typically designed to convey the losses of life or of healthy time consequent to disease onsets. These measure were usually based on life table methodologies, e.g., years of life lost (mortality only) and the quality-adjusted life year (QALY) or the DALY (incorporating mortality and morbidity).

### Statements and claims

Each statement retrieved could include more than one eligible claim. For example, three claims were taken from the text below.

*"Stroke is the third leading cause of death and a major cause of disability in the United States. In 1999, 167,366 deaths in the United States resulted from stroke. Approximately 30% of stroke survivors are permanently disabled and 20% require institutionalized care. Stroke is also a huge financial burden for patients, their families, and the health care system. The cost of stroke in the United States in 2002 is estimated to be $49.4 billion, which includes direct health expenditures and lost productivity resulting from morbidity and mortality." *[3]

These claims were classified as follows:

"Stroke is the third leading cause of death" - classified as an objective (rank) measure. "167,366 deaths in the United States resulted from stroke" - classified as an objective (count) measure.

"Approximately 30% of stroke survivors are permanently disabled and 20% require institutionalized care" - Two objective (proportional) measures, admittedly with an implied time dimension.

To provide a broader overview of the extent of uptake of the DALY, we searched MEDLINE for all citations of the DALY over the 16 years from its introduction in 1993 until 2009. The search was conducted using the following search terms:

1. "disability adjusted life year*"

2. DALY*

* Asterisks refer to a wildcard search operator

The term "DALY" in the author and address fields was excluded from the search. The titles and abstracts of the retrieved articles were scanned and categorized according to the ways the DALY was employed in the analysis. We found that the context in which the DALY was used could be categorized into three broad groups: 1) *economic and evaluative research*, in which the main purpose of the paper was to estimate a cost per DALY or to assess a technology or intervention in terms of DALYs averted; 2) *assessment of the burden from one or more diseases*, where the research was focused upon estimating the amount of disease in a population; and 3) *methodological*, where attention was focused on the strengths and weaknesses of the metric.

## Results

**Figure 1:**
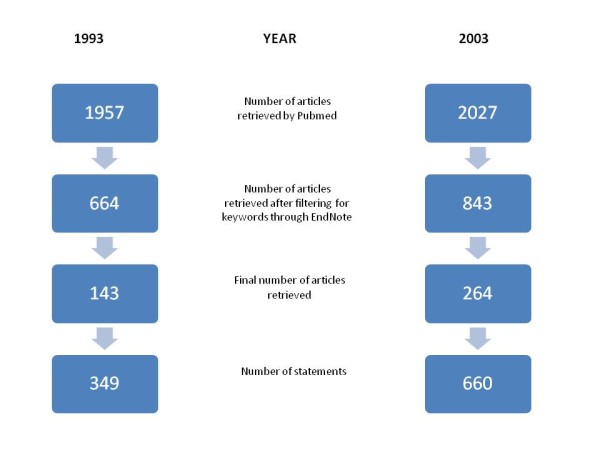
**Schematic representation of articles retrieved from three leading medical journals through a MEDLINE search and the number of articles resulting from the selection process**.

We found 143 papers in 1993 and 264 papers in 2003 that made one or more eligible claims in their introductory sections, yielding 349 claims in 1993 and 660 in 2003 (Figure 1).

The majority of the claims in 1993 and 2003 used objective metrics, including counts and proportions, to express the importance of the disease of interest. Some also used risk estimates and relative terms that stated disease effects in a population in relation to other diseases or from the same disease in other populations. Economic quantities, such as cost of treatment, were used in fewer than 5% of the claims, while time-based metrics were rarely employed. In 1993, one article expressed total years of potential life lost (YPLL) in the US and indicated the proportional contribution of different diseases and conditions to this total. In 2003, one article used the DALY to express the burden of cardiovascular disease in more and less developed countries, while another employed the QALY in four statements to compare the relative cost effectiveness of surgery versus medical therapy for people with severe emphysema. Most of the objective measures used were proportions and counts, while a substantial number were rates and rank measures. There was no significant change in the number of rates, risk, and relative measures used between 1993 and 2003 (p-value = 0.99) (Table 1).

**Table 1:** Distribution of the different types of metrics

Measure	1993 (n = 349) N (% of all)	2003 (n = 660) N (% of all)
**Objective metrics**	**336 (96.5)**	**628 (95.2)**

Proportions	126 (36.3)	264 (40)

Counts	102 (29.4)	143 (21.7)

Rates	47 (13.5)	118 (17.9)

Rank	38 (10.7)	54 (8.2)

Relative measures	13 (3.9)	42 (6.4)

Risk	10 (2.9)	7 (1.1)

**Economic metrics**	**12 (3.5)**	**26 (3.9)**

**Time-based metrics**	**1 (0.2)**	**6 (0.9)**

Those claims that expressed the importance of a problem by rank were most likely to use cause of death or membership in a grouping of diseases as the basis for ranking, e.g., "Lung cancer is the most common form of cancer" (Table 2). Very few authors ranked the importance of their disease as a cause of morbidity or by time-based measures.

**Table 2:** Distribution of the different types of ranking measures

	1993 (n = 36) N (% of all ranked)	2003 (n = 53) N (% of all ranked)
Cause of death	18 (48.6)	19 (34)

Cause of morbidity	1 (2.7)	4 (7.5)

Cause of disease	7(18.9)	5 (9.4)

'Most common form of...'	7 (18.9)	20 (37.7)

Burden/years of life lost	1 (2.7)	3 (5.7)

### PubMed search for DALY

**Figure 2:**
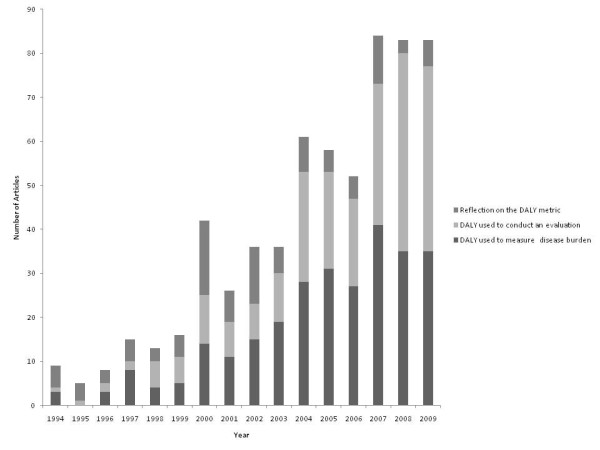
**The number of journal articles citing the DALY and the nature of its use from 1994 to 2009**.

The number of journal articles using the DALY metric increased gradually from 1994, reaching a maximum of 85 citations in 2007. A total of 279 papers over the 16 years were concerned with directly assessing the burden of one or more diseases. Alternatively, the DALY was used to express the burden of a disease of interest as background to the research presented. More than one-third of all papers (242 out of a total of 627 papers) used the DALY in the context of evaluations. While the number of DALY citations in burden assessments has been steady at an average of 30 articles a year, citations using the DALY metric for economic analyses have seen an increased use over recent years (Figure 2). The *Bulletin of the World Health Organization *was a frequent location for papers using the DALY. Citations that reflected on the use or calculation of the DALY peaked in 2000. Prior to and after 2000, such papers averaged about four per year and seven per year, respectively.

As a proportion of the total number of records in PubMed, total DALY citations ranged from 0.1% in 1994 to 0.97% in 2009, showing a similar pattern of rises and falls in different years as seen in Figure 2 (data not shown).

## Discussion

In the materials reviewed, over the time period of interest, we found that there has been limited use of time-based metrics when making claims about the importance of diseases. This was not because the metrics actually used were always adequate for the purpose at hand. For example, counts were often used even though this metric left the reader with little guidance on how to interpret the magnitude that was being conveyed (i.e., is 100,000 road traffic deaths a large or small number?). The import of different rates of change may also be challenging for the reader.

Why then is there little evidence of a trend toward time-based metrics for conveying disease burdens? There are several possibilities:

• When making claims, researchers may not consider the choice of metric to be a matter of any consequence.

• Researchers may be unconvinced of the relative merits of time-based versus event-based metrics for this purpose.

• Journal editors may not favor the use of time-based metrics.

A number of studies have demonstrated that the rank position of a disease can be very sensitive to the measure used [4-7]. Policymakers and others seeking information for prioritization purposes will be better served if more attention is paid to choosing metrics that are appropriate to the purpose at hand. Although it is unlikely that the claims made in the introductory section of scientific reports will be used to make important policy decisions, the apparent lack of attention afforded these claims may be indicative of a broader neglect afforded to the choice of metrics when expressing disease burden. "All measures of population health involve choices and value judgments in both their construction and their application" [8]. This is as true for seemingly simple metrics such as death counts as it is for complex summary measures like the DALY.

One limitation of the first part of this study is that we restricted our analysis to three leading general medical journals. Use of time-based measures may be more prominent in specialized journals. Indeed, in our broad search for citations of the DALY, 23 articles picked up from statements in the introductory section of articles were commonly from parasitology and mental health journals as well as the *Bulletin of the WHO *(data not shown). Our interest here, however, was in assessing the spread of the practice, and leading general journals were considered an appropriate starting point.

The second part of our study assessed trends in the uptake of the DALY itself in the whole of the scientific literature indexed in MEDLINE. DALY usage itself has seen some increased use over the past decade. A second limitation of this study concerns the search terms used for this analysis. Our search was limited to the terms "DALY" and "disability adjusted life years." A broader search would have extended the terms used to include such phrases as "burden of disease." Some authors, for example, rather than report DALYs directly, may represent their calculations as a proportion of the total burden. On the other hand, "burden of disease" does not necessarily refer to burden as measured by the DALY or any other time-based metric. Furthermore, in many publications, it is not clear what is implied by burden. For these reasons, and for the purposes of this paper, it was felt that an analysis of direct citations of the DALY would provide an adequate understanding of the utilization of the concept rather than the broader and opaque concept of burden.

Two points about the DALY are interesting to note. First, much of the increase was in the context of economic assessments. Second, critical engagement with the metric and related metrics peaked in 2000 and has since stagnated. This seems to suggest that the DALY has found a niche where its application is seen to be less controversial.

This paper does not aim to suggest that the DALY, or any other time-based metric, is the only (or even the more) suitable metric for the purpose of informing policy. What we have hoped to demonstrate is that the DALY and other time-based metrics, which were designed for the purpose of expressing burden of disease, have not been taken up in common contexts where it is desirable to communicate the burden of disease effectively. The use of the DALY is well-known to be controversial and accompanied by many technical and theoretical concerns, and this may be a reason for researchers' apprehension. We have shown here, however, that the current practices in expressing disease burden in the context of opening statements in journal articles are also limited, and metrics employed are often not intuitively informative. In other words, the choice of metric for expressing the burden of disease should be more deliberate, choices should be carefully considered, and reflection on this topic should not stagnate.

## Competing interests

The authors declare that they have no competing interests.

## Authors' contributions

JP conceived of the study, HG and JP designed the study, and HG analyzed the data and drafted the article. JP revised the draft. HG and JP read and approved the final manuscript.
